# An Overview of Methods for Cardiac Rhythm Detection in Zebrafish

**DOI:** 10.3390/biomedicines8090329

**Published:** 2020-09-04

**Authors:** Fiorency Santoso, Ali Farhan, Agnes L. Castillo, Nemi Malhotra, Ferry Saputra, Kevin Adi Kurnia, Kelvin H.-C. Chen, Jong-Chin Huang, Jung-Ren Chen, Chung-Der Hsiao

**Affiliations:** 1 Master Program in Nanotechnology, Chung Yuan Christian University, Chung-Li 320314, Taiwan; fiorency_santoso@yahoo.co.id; 2 Department of Bioscience Technology, Chung Yuan Christian University, Chung-Li 320314, Taiwan; ferrysaputratj@gmail.com (F.S.); kevinadik-adi@hotmail.com (K.A.K.); 3 Department of Bioinformatics and Biotechnology, Government College University Faisalabad, Punjab 38000, Pakistan; smalifarhan@gmail.com; 4 Faculty of Pharmacy, The Graduate School and Research Center for the Natural and Applied Sciences, University of Santo Tomas, Manila 1008, Philippines; alcastillo@ust.edu.ph; 5 Department of Biomedical Engineering, Chung Yuan Christian University, Chung-Li 320314, Taiwan; nemi.malhotra@gmail.com; 6 Department of Applied Chemistry, National Pingtung University, Pingtung 900391, Taiwan; kelvin@mail.nptu.edu.tw; 7 Department of Biological Science & Technology College of Medicine, I-Shou University, Kaohsiung 82445, Taiwan; 8 Center of Nanotechnology, Chung Yuan Christian University, Chung-Li 320314, Taiwan

**Keywords:** zebrafish, cardiac physiology, heart rate, detection method

## Abstract

The heart is the most important muscular organ of the cardiovascular system, which pumps blood and circulates, supplying oxygen and nutrients to peripheral tissues. Zebrafish have been widely explored in cardiotoxicity research. For example, the zebrafish embryo has been used as a human heart model due to its body transparency, surviving several days without circulation, and facilitating mutant identification to recapitulate human diseases. On the other hand, adult zebrafish can exhibit the amazing regenerative heart muscle capacity, while adult mammalian hearts lack this potential. This review paper offers a brief description of the major methodologies used to detect zebrafish cardiac rhythm at both embryonic and adult stages. The dynamic pixel change method was mostly performed for the embryonic stage. Other techniques, such as kymography, laser confocal microscopy, artificial intelligence, and electrocardiography (ECG) have also been applied to study heartbeat in zebrafish embryos. Nevertheless, ECG is widely used for heartbeat detection in adult zebrafish since ECG waveforms’ similarity between zebrafish and humans is prominent. High-frequency ultrasound imaging (echocardiography) and modern electronic sensor tag also have been proposed. Despite the fact that each method has its benefits and limitations, it is proved that zebrafish have become a promising animal model for human cardiovascular disease, drug pharmaceutical, and toxicological research. Using those tools, we conclude that zebrafish behaviors as an excellent small animal model to perform real-time monitoring for the developmental heart process with transparent body appearance, to conduct the in vivo cardiovascular performance and gene function assays, as well as to perform high-throughput/high content drug screening.

## 1. Why Zebrafish Behavior Is a Good in vivo Model to Address Cardiac Physiology and Toxicology?

Zebrafish have been widely used as an in vivo model for toxicology evaluation. Toxicity screening may include information on the potential off-target effects of target compounds on the cardiac system, central nervous system, intestinal tract, auditory and visual functions, and bone formation. Cardiotoxicity is one of the major concerns in toxicological studies. It is associated with potential QT interval prolongation, an indicator of fatal cardiac side effects in the heart’s electric cycle [1]. The use of zebrafish has been reported to be very reliable, describing the potential toxicity of drugs to the human cardiovascular system [2]. Human cardiovascular disorders have been recapitulated in zebrafish genetic models [3]. Moreover, numerous human cardiovascular drugs have demonstrated similar effects on zebrafish physiology. It is reported that humans’ cardiac electrophysiology is more similar to that in zebrafish than in rodents [4]. A previous study has shown that more than 95% of drugs that induce QT prolongation in humans have similar effects in zebrafish [5]. Effects of chemicals on zebrafish cardiac development have been reported in a study by Incardona et al. [6], Transient changes in zebrafish cardiac conduction have been observed after exposure to Polycyclic Aromatic Hydrocarbons (PAHs), which are pollutants in the aquatic environment. The result shows no effect on the initial stages of cardiac loop formation. However, significant changes occur after loop formation, including the development of cardiac valves, formation of trabeculae, and the thickening of the ventricular myocardium. Additionally, biological characteristics, ease of genetic manipulation, facilitation of chemical screening, and genetic similarity to humans are features that contributed to zebrafish as a versatile animal model for cardiovascular research. Previously, zebrafish have been generated to study lipid metabolism and hypercholesteremia, major risk factors for cardiovascular disease [3]. Furthermore, Chen et al. reported mutations in zebrafish, useful for in-depth cardiac development research [7]. These advantages make zebrafish a suitable animal model for cardiac physiology and toxicity studies.

Zebrafish’s hearts consists of two chambers, an atrium, and a ventricle, which has a similar pumping mechanism to mammals’ hearts in cellular and molecular levels [8]. These similarities include the flow of the blood from sinus venosus into an atrium; the blood moves through the ventricle to the aorta as controlled and directed by the heart valves; has specialized endocardium musculature that drives a high-pressure system; the regulation of heart rhythm controlled by electrical current that flows through the heart, causing the muscles to contract and blood pumped out; and a pacemaker that discharged the electrical current determines the heart rate [9]. Moreover, the zebrafish heart rate was reported at 140–180 bpm, where it is much closer to the human fetal heart rate (130–170 bpm), which is far slower compared to mouse heart rate (300–600 bpm) [10].

As a human cardiovascular disease model, adult zebrafish can be applied to study damage of myocardial tissue caused by prolonged ischemia and hypoxia, which is typical of myocardial infarction and a leading cause of heart failure. Adult zebrafish hearts regenerate, while adult mammalian hearts do not. Adult zebrafish are reported to have the robust regenerative ability in the heart muscle [11]. They can regenerate their ventricles without having a visible scar when surgical amputated or cryo-injured. Moreover, both atrium and ventricle can regenerate in response to genetic cardiomyocyte ablation, even if the ablation is widespread enough to initially cause clinical heart failure [12]. The cardiovascular system is also linked to the zebrafish swimming stamina. Generally, there are a 20 to 50 beats per minute increase in heart rate associated with swimming activity as early as day 5, suggesting that neural or hormonal mechanisms required for heart rate acceleration are functional in early development. Alteration in the heart rate is closely related to the cardiac output. A lower heart rate could reflect lower cardiac output for any given swimming speed in the high stamina group, where this phenomenon could be comparable to athletes [13].

## 2. Overview of Embryo Cardiac Rhythm Detection

Zebrafish embryos have been widely used to understand heart development and physiology, due to their body transparency, small body size, high fecundity, rapid developmental process, and similar heart structures as humans. These are some features that help scientists to understand the mechanisms in mammals [14]. In addition to transparency, small body size and high fecundity make embryonic zebrafish a fitting animal model in a high throughput heart rate assay. The assay scored 229 (95%) heart rates out of 240 arrayed embryos per 96-well plates, and the heart rates were scored accurately [15]. Other advantages of using zebrafish embryo are the ease of genetic manipulation which can also facilitate identification of the function of human mutations [16] and its ability to survive for several days (4–5 days) without active circulation, providing enough time to study the defects and to dissect cellular and molecular mechanisms [10]. Zebrafish heart development can be traced back to 5 h post-fertilization (hpf) during which cardiac progenitor cells were originated. These cells will migrate to reside in the posterior half of the anterior lateral plate mesoderm (ALPM) during the gastrulation process at 15 hpf. At 24 hpf, heart tube will form and initiate cardiac contraction [17]. Finally, the heart loop from the right-sided ventricle and left-sided atrium can be observed at 48 hpf, although major heart structures have been formed, the heart is still immature and lacking functions for supporting future growth [16]. Regarding this information, zebrafish embryo at 48 hpf can be used to see the whole heartbeat frequency and early-stage chemical treatment as early as 3 hpf can be performed to observe developmental effects [10].

There are several methods to assess cardiac functions in zebrafish embryos, including the manual counting from slow-motion replay in the video, micro pressure systems, microscope techniques, and electrocardiogram devices. However, using electrocardiographic signals in a zebrafish embryo requires precise positioning of the electrodes to obtain reproducible signals [18]. Kang et al. found outthat counting heartbeats manually was more unreliable as there is a high possibility of biased results due to individual error, which measures the variation between repeated experiments by an individual, and from the crowd error, which measures the variation between different operators, even when the counting was done by trained individuals [19]. Gaur et al. [20] also reported the manual counting method. Three independent researchers counted the heartbeat from 10-second length video and, validation was also performed by exposing the zebrafish embryo to Isoproterenol, a well-known beta-adrenergic agonist, which increases heart rate. Their results showed that manual counting is unreliable and may lead to inaccurate data due to the variably experienced researchers.

In overcoming the drawback of manual analysis, several approaches have been developed to monitor cardiac function in zebrafish, such as automated and semi-automated based on computer-controlled, image-based heartbeat detection techniques. Fink et al. [21] and Ocorr et al. [22] outlined a semi-automatic technique of analyzing heartbeats based on the variation of intensity changes over a set of pixels selected by a manually provided threshold, and by stacking a manually selected vertical slice containing the ventricular wall. Other research manually selected the Region of Interest (ROI) before performing an automatic method of characterizing the cardiac function in zebrafish embryos [23]. On the other hand, for the sake of time efficiency, automatic counting was then developed [18,21,24,25,26]. The development of automated algorithms for estimating heart rate in zebrafish larvae begins with the automatic determination of a suitable ROI and heart rate calculation [25]. Comparison of previously published method to detect cardiac rhythm in zebrafish larvae can be found in Table 1.

## 3. Commercial or Third-Party Software

Normally, heart rate assays in zebrafish embryo are performed on one embryo per video/imaging field [9,27]. The high-throughput method from multiple zebrafish embryos per imaging field was established [32]. FisHRateZ, a LabVIEW-based program, can measure the heart rate of multiple zebrafish embryo per imaging field. By utilizing an algorithm to locate the heart automatically and derive average pixel intensity vs. time, data were generated from each embryo’s cardiac cycle. FisHRateZ increases the throughput and reliability of the cardiotoxicity screening method. This method is the first approach to detect and place ROI on multiple zebrafish up to five embryones/well. However, the algorithm is unable to assign precise ROIs on overlapping embryos. Therefore, this study suggests that analysis might provide better results with two embryones/well [32]. Another commercial software is MetaXpress. It is a high content image analysis software that uses interactive semi-automated journal scripts to isolate the region of interest and quantify heart rate, circulation, pericardial area, and intersegmental vessel area. Moreover, a fully automated journal script has been used to quantify body length. The main principle of MetaXpress is to detect the heart rate based on intensity variation [33]. SoftEdge Myocyte Cell Length Acquisition Module, developed by IonOptix Corporation (https://www.ionoptix.com/), provides an edge-detection system to evaluate systolic and diastolic by using automatic video. The edge detection is based on either image intensity or the derivative of image intensity. The software analyzes each video image and treats the crossing of the threshold values as the edge location [35]. RvVisualPulse software developed by Zgenebio and RasVector Technology in Taiwan (https://www.zgenebio.com.tw), can simultaneously track the cardiac rhythm in both atrium and ventricle. This software’s basic principle is also based on dynamic pixel change and very convenient for potential arrhythmia detection in zebrafish. Another two third-party software called MicroZebraLab from Viewpoint company (http://www.viewpoint.fr/en/home) and DanioScope from Nodules company (https://www.noldus.com/danioscope) also can be used for cardiac rhythm measurement with the advantages of easy operation.

## 4. Dynamic Pixel Changes Method

Matlab is one of the software commonly used to measure heart rate in zebrafish embryo [18,21,24,25,26]. Detection and quantification of movement can be performed by algorithms written in Matlab. The Frame Brightness Algorithm tracks changes in average light intensity of each frame combined with a Changing Pixel Intensity Algorithm to detect movement by comparing the intensity changes in individual pixels from one frame to the next. During heart contraction, frame brightness decreases in correspondence to darker pixels. As the heart muscle contracts, the cell membrane becomes more concentrated and obscures more of the transmitted light [21]. A similar approach also has been taken by other researchers [24,25,26]. However, the Matlab programs were too complicated for researchers who did not have a computer programing language background.

To overcome the scriptwriting requirement, the open-source software of ImageJ, initially developed by the National Institutes of Health (USA), is a public domain, Java-based image processing program [36]. The ImageJ platform is adaptable with no requirement of scriptwriting to execute the program. Similar to Matlab’s approach, the cardiac rhythm measurement method is based on the dynamic pixel changes in the heart region. Sampurna et al. [9] proposed an image-based method that did not require transgenic fish or expensive instruments to detect heart rate. Only a conventional dissecting microscope and a less expensive charged-coupled device (CCD) were needed. The highest peak rhythm represented the greatest abundance of red blood cells pumped out of the heart chamber is defined as a contraction (systolic phase). On the other hand, the lowest peak rhythm represented the greatest abundance of red blood cells, which filled the heart chamber and was defined as relaxation (diastolic phase). In previous studies, zebrafish heartbeat calculations mainly focused on the whole heartbeat. The atrium and ventricle beating rhythm was not studied in detail. In the method proposed by Sampurna et al. [9], the potential drugs for atrioventricular blockage could be studied in detail by the image-based method. However, this ImageJ-based method performed manual counting, which involved multiple steps before obtaining cardiac function. Gaur et al. [20] combined ImageJ with an algorithm written in Microsoft Excel and an online Google spreadsheet. Instead of using Origin Pro software to calculate the heartbeat from the dynamic pixel pattern, the algorithm could be used in smoothening raw heartbeat data, and automatic peak detection to avoid unambiguous peak detection [20].

## 5. Indirect Measurement from Blood Vessels

The heartbeat in zebrafish embryos could be detected indirectly from its blood cells. Shuk Han Cheng’s team at the University of Hong Kong described a non-invasive technique that integrated digital motion analysis and power spectral analysis to determine heart rate and heartbeat regularity in peripheral blood vessels. Pulsatile movement of blood cells was observed in the caudal vasculature of zebrafish embryos. A waveform of dynamic pixels can visualize the posterior cardinal vein (PCV) and blood vessels’ oscillatory movement. The heart rate was determined by digital motion analysis and power spectral analysis by extractingthe cardiac rhythm’s frequency characteristics. This study showed that the program could also detect the changes in the variation of heart rate in zebrafish embryos after exposure to Terfenadine, a known QT-prolonging drug [34]. Heartbeat regularity is an essential parameter for cardiac function and is associated with cardiotoxicity in human beings.

On the other hand, another study proved that the heart rhythm calculation from blood flow velocity could be done using the ImageJ-based method [27]. The dorsal aorta (DA) vessels are directly connected to the heart chamber, making the DA regional blood flow pattern following the ventricle’s pumping. Similar to the previous study, the calculation is based on pixel intensity change. The principle of pixel intensity change is based on the cell numbers and differences in the degree of an object affected by speed. During heart pumping, in the DA, the highest peak of blood flow pattern represents the systolic phase, where the dynamic pixel change intensity would be higher, and a higher peak rhythm is formed. On the other hand, at low blood flow velocity, a lower peak rhythm is formed. The study found that the time interval of blood flow patterns to be in agreement with heart rate. Heart rate measurement could also be done indirectly based on the recorded blood flow video. However, heart rate measurement calculated from blood flow patterns may lead to biased results [27]. According to Grimm and colleagues (1996), the variability of heartbeat interval could be determined not only from ECG but also from the blood pressure. Their results showed that waveform data derived from the blood pressure was consistent with the data from ECG [37].

## 6. Kymograph-Based Method

Kymograph is a two-dimension (2D) plot containing time and space information created from time-lapse images of the region of interest. It can quantify the movement velocity and trajectory of the objects. In measuring velocities based upon kymographs, the moving object’s path has to be marked [38]. A kymograph is generated from the fluorescence intensity, presented along the segmented line (*x*-axis) over time (*y*-axis). Diagonal lines in kymograph demonstrate moving objects over time, and ventricle lines illustrate stationary objects [39]. In zebrafish, a kymograph was used to quantify heart rhythm. The results of kymograph-based quantification of the cardiac cycle could illustrate bradycardia effect and irregular heart rhythm in *Islet-1*(*isl1*) mutant zebrafish embryo, as the distance between two dotted lines encompassing a full cardiac cycle is much longer in the mutant than in the sibling [40]. Other research used kymograph to record the frog’s myocardial activity [41].

Kymographs have long been used for visualizing intracellular motion. Kymographs can be generated from a time-lapse image sequence using image analysis software, such as MetaMorph (Molecular Devices), NIS-Elements (Nikon), and ImageJ (NIH). By using the ImageJ, the kymographs can be plotted, smoothened, and quantified using the Smoothed Plot Profile and Find Peaks tools provided in the BAR Plugin [42]. The advantage of a kymograph is time-lapse data compressed onto a single image allowing visualization of the motion. For another, the human eye is very good at picking outlines, even in substantial noise. With kymographs’ help, it can be much easier to see how many moving particles are present [43]. Freely available kymograph software has been used to visualize live primary cilia dynamics using fluorescence microscopy and in quantitative measurements of intraflagellar transport (IFT) movement within cells [43,44]. Thus far, the implementation was limited in zebrafish and other aquatic animals. Studies on cardiac rhythm detection by using the kymography method can be explored in the future.

## 7. Laser Confocal Scan Method

A laser-scanning confocal microscope is a sensitive and precise tool for measuring cardiovascular changes in the zebrafish embryo. It is also reported that the measurements are similar to those obtained with Doppler echocardiography [45]. A previous study reported that the heart rate evaluation in transgenic larvae could be obtained from sequential images acquired by a resonant laser scanning confocal microscope. The heart region extracted was based on fluorescence (red/green), and was processed further by morphological operations, based on the threshold values [18]. Another technique, called laser-scanning velocimetry, provided continuous blood cell velocity measurement necessary for estimating cardiac output and other cardiovascular function parameters. Line scans are images constructed with laser-scanning microscopy by repeatedly imaging one pixel-wide line. When the scan line is placed across a vessel, either perpendicular or parallel to blood flow, the resulting line scan image contains information about circulation dynamics. The continuity of velocity information provided by laser-scanning velocimetry makes it possible to measure blood acceleration and deceleration, which are similar parameters that are attainable using Doppler echocardiography [45]. However, this method is only applicable to transgenic embryos with fluorescence-labeled blood cells and requires costly instrumental settings.

## 8. AI Deep Learning-Based Method

Integrating artificial intelligence (AI) into cardiovascular research shows optimistic promise in generating quantitative diagnostic for clinical implementation. AI integration is not a simple transition since machines replace humans. However, AI could help researchers and clinicians to make better decisions. AI can integrate complex omics data with additional layers of information, including imaging and electronic health data, to provide accurate information and quantitatively analyze large data sets, which would not be feasible manually [46]. Several effective automated heartbeat detection methods have been developed to reduce the workload for larva heartbeat analysis. A previous study utilized a deep learning platform, called Cardiac Functional Imaging Network (CFIN) to design automatic and rapidly segment atrial and ventricular boundaries in live zebrafish embryo hearts and utilize data to calculate other cardiac function parameters. This aspect of CFIN was built in Matlab’s underlying architecture of the deep convolutional encoder-decoder SegNet. As a result, CFIN is capable of detecting elevation in cardiac output and sensitivity over fractional shortening. This method was shown to be a powerful new tool to accurately measure heart function in a vertebrate model organism [30]. Custom Matlab-based software, called FishInspector, was created to detect morphological features in zebrafish embryos automatically. The zebrafish heart, as the region of interest (ROI), is identified by comparing the absolute difference in pixel intensity between two consecutive frames. However, this method depends heavily on image quality (camera and microscope settings, resolution, contrast, intensity) [47]. Another automatic method, Zebrafish Heart Rate Automatic Method (Z-HRAM), was developed to detect and track the heartbeats of immobilized, ventrally positioned zebrafish larvae without direct heart observation. This method is well suited for the analysis of low resolution and low-frequency image data. Z-HRAM focused on detecting the body deformation, and computation of the pixel-wise motion images of all images in the video recording to associate zebrafish body deformation frequency with the heartbeat. Heartbeats detected from Z-HRAM were shown to correlate reliably with those determined through corresponding electrocardiogram and manual video inspection. However, Z-HRAM is currently limited to the detection of heart rate and beat-to-beat intervals [31].

In a deep-learning-based method, training was necessary to demonstrate consistency with the overall assessment and compensate for the automated image analysis [47]. Xing et al. [31], conducted training at a variable learning rate beginning at 0.1, which was reduced by a factor of five every two epochs. Using these parameters, they observed an increase in accuracy and decreased in function for both the training and cross-validation datasets, which were consistent with successful training.

## 9. Electrocardiography Method (ECG)

The optimization of ECG detection in early stage zebrafish larvae has been demonstrated in its utility and detecting the effect of QT-prolonging. Anesthetized zebrafish embryos were transferred to paraffin wax. The glass micropipette was then positioned on the skin surface between the ventricle and atrium with no penetration. As early as 2 dpf (days post-fertilization) of zebrafish could be used for ECG measurements, as the compound action potentials have been generated, and effective cardiac conduction are already present [48]. Electrical Potential Sensing (EPS) technology was established to record in vivo electrocardiogram activity from the heart of the zebrafish embryo at 3 dpf and 5 dpf. This method does not require complex post-processing tools, and importantly, the embryo is maintained alive. The EPS sensor uses a metallic titanium (Ti) based central electrode coated with a titanium dioxide (TiO_2_) film/membrane acting as a dielectric [49]. Nonetheless, ECG methods were considered challenging to record heartbeat in zebrafish embryo due to its small size [18]. Moreover, the requirement of using expensive specialized devices and software has kept it inaccessible to many scientists.

## 10. Overview of Adult Cardiac Rhythm Detection

The cardiac rhythm detection method in adult zebrafish (2–3 months old) is more complicated than techniques applicable to the embryonic stage due to a loss of transparency and dependence on anesthetic drugs. In aquatic toxicity studies, the cardiovascular function has been an indicator of changes in the physical condition of health or stress in response to an environment [50]. Zebrafish is an excellent model for its sensitivity to drug treatment. It can reveal a decreased in heart rate concerning the human *ether-a-go-go* gene (hERG) channel blockade, allowing for drug testing. Moreover, zebrafish also provide an efficient genetic approach to reveal the genetic basis underlying molecular mechanisms of numerous heart diseases [51]. It could be a robust human cardiac electrophysiology model since its heart rate, and action potential morphology are similar, although some differences are apparent [52]. Moreover, adult zebrafish exhibit the amazing regenerative capacity of heart muscle [11], thus making zebrafish a promising model to study cardiovascular disease in humans.

**Figure 1:**
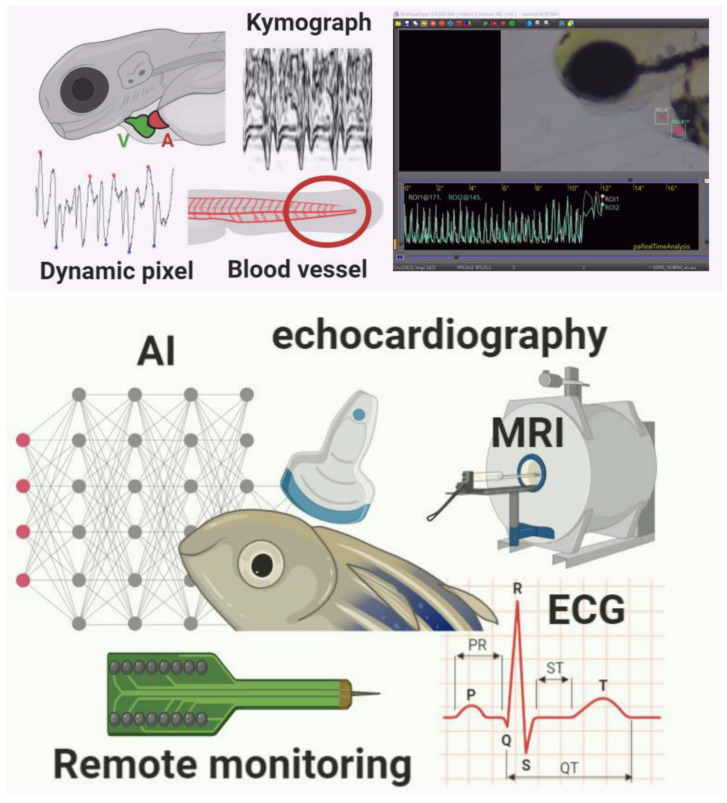
Summary of different methods used to measure cardiac rhythm in either embryo (upper red panel) or adult (bottom green panel) zebrafish. Several methods based on either dynamic pixel change or kymograph are proposed in the embryonic stage of zebrafish. Some commercial third-party software is also available for cardiac rhythm measurement. In adult zebrafish, some instruments like Electrocardiography (ECG), Echocardiography (Echo), and Magnetic Resonance Imaging (MRI) can be used for cardiac rhythm measurement.

Advanced imaging techniques are required to measure all facets of cardiac function in adult zebrafish. Electrocardiogram (ECG) is a standard diagnostic tool to detect cardiovascular disease in humans. The body surface ECG recording of adult zebrafish was first developed in 2006 by Milan et al. at the Cardiovascular Research Center, Boston, MA, USA [53], followed by several modifications made in the methods of ECG recording in zebrafish. ECG waveforms present a distinct P wave, QRS complex, and T wave. It is reported that the waveform in adult zebrafish is comparable with the waveform presents in humans [54]. High-frequency ultrasound imaging has been proposed as a suitable tool for achieving high-resolution imaging of adult zebrafish tissue structures [55]. The use of echocardiography with a high frequency (50–70 MHz) probe can allow for high resolution, real-time, non-invasive imaging to examine many cardiac structure parameters and function [52]. Magnetic Resonance Imaging (MRI) is also well-established for imaging adult zebrafish as a cardiovascular disease animal model. This method could provide three dimensional (3D) live images to depict the embryonic heart developmental processes in zebrafish and the injury recovery of adult zebrafish skin. However, due to the small size of zebrafish, a high-resolution micro-imaging magnet was required [56,57]. Modern electronic sensor tags were recently established to collect high-resolution data on the movement and physiology of zebrafish. The loggers were surgically inserted into the fish, close to the heart. The application of the loggers is considered difficult or even challenging due to the minuscule scale surgical procedure. Moreover, it may cause stress on the fish. New cordless heart rate bio-loggers were introduced to provide easier tag-deployment and surgery, hence reducing stress for the fish [58,59]. With all the methods mentioned, the advantage of using aquatic creatures like fish is that they can be easily accommodated in a smaller space. Nonetheless, at the same time, the smaller size restricts the researcher from performing several activities, unlike other vertebrates, which allow manipulation with ease [60]. Summary of cardiac rhythm detection method in larvae and adult zebrafish can be found in Figure 1 and the comparison beetwen previously published methods to detect cardiac rhythm in adult zebrafish can be found in Table 2.

## 11. ECG-Based Detection Methods

Electrocardiogram (ECG), including up to five embryones/well, has become the most extensively used non-invasive diagnostic tool for evaluating cardiac diseases. Measurement of ECG intervals is considered one of the most important and gold standard techniques because ECG interval provides an indirect assessment of the heart’s state and can be indicative of certain cardiac conditions. So far, ECG has been an excellent way to record the heartbeat in adult zebrafish with less harm to the fish. The results were also proved to be very accurate without a requirement for additional reading and formula [66]. In ECG, three components are crucial for detection: T wave, P wave, and the QRS complex. The P wave represents the depolarization of the atrium; QRS complex represents depolarization of the ventricle, and the T wave, represents the repolarization of the ventricle [67]. In addition, the QT interval is the most important time interval in the ECG waveform, defined as the time from the upstroke of the QRS complex to the end of the T wave. This interval represents the duration of the ventricle’s electrical activity (depolarization and repolarization) in a given heartbeat. The reported QT intervals of adult zebrafish in previous studies ranged from 250 ms to 600 ms with multiform ECG signals [54]. The overall ECG waveform can be seen in Figure 1. In establishing the authenticity of ECG signals in fish, several different recordings were taken. A study by Haverkamp et al. [68] showed that the average heart rate of the zebrafish is around 148 beats per minute; the QRS interval recorded was 44 ± 3 ms. The average PR interval was about 62 ms, and the RR interval was around 469 ms. Lastly, the QT interval recorded was 269 ± 60 ms. The results were consistent with the past ECG systems and paved the way for more complex types of ECG experiments.

Leor-Librach et al. [69] developed a computerized system for the controlled increase of heart rate by Isoproterenol based on a modified proportional-integral controller. Previous references and protocols were used to carry out some important steps for developing a pharmacodynamic model for model-based, adaptive control systems, and as a part of larger cardiovascular models [69]. In optimizing the adult zebrafish ECG method for assessing drug-induced QTc prolongation, specific procedures using the histamine receptor antagonist, Terfenadine as a test drug, were carried out for ECG recording in the adult zebrafish. The fish were anesthetized and mounted in a wet sponge, so thatthe upper side could be easily analyzed. The sponge had been cut into a triangle to preventing the water from infiltrating the gill. The scales which cover the heart were removed; thus the electrode could penetrate the skin vividly [63]. The dermis peeling on the chest significantly enhanced signal recording from the muscle layer on the chest. In addition, opening the pericardial sac further enhanced the ECG signal’s surface without changes to the electrophysiology [54]. The experiment’s region of interest was below the fishes’ fins, and it was constantly monitored through a microscope. The electrode responsible for recording the ECG signal was carefully inserted without damaging any tissue or muscle. The positive needle electrode was inserted between the pectoral fins. The negative electrode was placed in two-third of the body length down from the head and positioned near the anal region or reproductive cavity. Another grounding electrode, used as a reference electrode, was placed at the sponge’s left corner. The penetration depth of the needle electrodes was about 1–1.5 mm [63]. The pectoral electrode was constantly moved so that a stronger P wave, T wave, and QRS complex were clearly detected and recorded accurately. The timing of the experiment depended on the objectives and the response time for the ECG recording.

There are several limitations in the ECG acquisition systems as only a short period can be recorded, and it requires the use of an anesthetized animal [70,71]. Notably, the anesthetics could only sedate the fish for about 30 m, after which, it is needed to be transferred to a recovery tank [69]. These two factors could lead to the inconsistency of results. The appropriate type of anesthetic drugs and a precise dose should be carefully chosen to avoid unreliable results. The ECG is only suitable for manual measurement of fish, one at a time, which is another limitation when screening multiple fish. Moreover, long-term ECG monitoring with repeated experiments will stress zebrafish, and weak ECG signals generated [51].

T-wave is the most significant ECG wave that shows the cardiac cycle’s repolarization from beat to beat. ECG acquisition method improved with the development of microelectrode array (MEA) membranes that can provide an appropriate signal-to-noise ratio (SNR) with complete P wave features, QRS complex, and T-wave. This method has recorded ECG signals that exhibit a clear difference between injured heart muscle ECG and normal heart ECG reading [72]. The additional limitation for ECG recordings in adult zebrafish is in the application of drug discovery where the dosage of the drug could not be determined precisely, making the data difficult to interpret. Differences in acclimatization and flow rate of oral perfusion may differ significantly between various laboratories, adding the result’s high variances [63].

The lack of well-defined diagnostic parameters for specific-disease patterns is one of the big challenges in doing ECG recording. This makes the traditional methods of algorithmic identification even harder to do. Despite this, a trained eye can identify abnormal features, even when an algorithm fails. Therefore, the new current methods which make use of machine learning were proposed for ECG waveforms. In recent studies, computational analysis on surface ECG recording and Deep Neural Network (DNN) achieved much more accuracy in detecting the premature ventricular contractions than Artificial Neural Network (ANN). It has been established that ANN shows accurate results in small datasets, and both of the ECG analysis tools are based on Image processing techniques. Zebrafish ECG images can be transformed in Matlab software to the digitized values as in Human ECG. Most of the experiments performed on awake zebrafish compared to anesthetized fish and artifacts dominated the raw signals. Moreover, the noise was removed by using Wavelet transform [51].

### 11.1. Noise Interfaces for ECG

The ECG recording methods used in adult zebrafish showed similar noise interference as in human ECG recording. However, the high noise level inherent in the signal is an essential challenge to identify ECG recording in adult zebrafish. The noise sourced could be influenced by the power line artifact, electrode contact noise, and muscle movement [73]. A protocol has been proposed for noise-free signals propagation and obtaining raw ECG signals without any data preprocessing as a wavelet transform. Thus, making the characteristic peaks of the P wave, QRS Complex, and T-wave easily identifiable [54].

The significance of the conventional method used in practice in ECG recording of adult zebrafish has been incorporated with of ECG analysis efficiency using an electrophysiology software package. ECG recording in zebrafish has a high-frequency ECG signal, which varies in a range of 50 Hz power line interference. The components are having low frequency exhibit muscle and movement artifacts. It has been demonstrated that pericardial sac with the dermis and silvery affect the ECG recording of adult zebrafish as zebrafish dermis is densely composed with a pack of collagen fibers [74].

EZ-Instrument Technology Co, Taiwan, has developed a simple ECG kit for teaching and research to make the extent of recording in zebrafish easy and simple. The zebrafish ECG kit comprises an integrated signal receiver and amplifier with the software package used for data processing. The specialized electrodes were also designed for anesthetized adult zebrafish. The design of three needlepoint electrode probes is comprised of the pectoral, abdominal, and grounding electrodes [56]. In reducing the noise in an aqueous environment, insulating paint was used on the electrodes’ surface with a harbored stainless-steel needle. The area of signal detection was defined to 1 to 1.5 mm on the head of the needle. The tail of the needle was welded with the connecting wire having a 3-pole auxiliary connector cluster. Zebrafish ECG recording by this method exhibited two micromanipulators that hold the pectoral and abdominal needles and a computer system with the software package for the analysis [75].

It must be noted that the background noise was more pronounced in adults than the optically transparent embryos. Therefore, appropriate normalization and validation of data may be required for comparative purposes. Acclimatization for 16 h in a recirculation system then 2 h of acclimatization in a semi-static tank used to immobilize the fish before recording ECG was found to be the best acclimatization time to produce consistent ECG signal. Regular, stable, reproducible, and noise-free ECG with nearly similar R–R intervals were considered consistent ECG [63]. Grounded Faraday cage was commonly used in some methods to minimize the background noise [48,51,63,76]. On the other hand, in a previous study, background noise was effectively reduced by applying the optimum signal threshold and Savitzky-Golay smoothing. The Savitzky–Golay algorithm also allows unbiased smoothing and real-time data calculation without a need for noise statistics [61].

### 11.2. Comparison of Human ECG with Zebrafish ECG and Application for Toxicity Assessment

**Figure 2:**
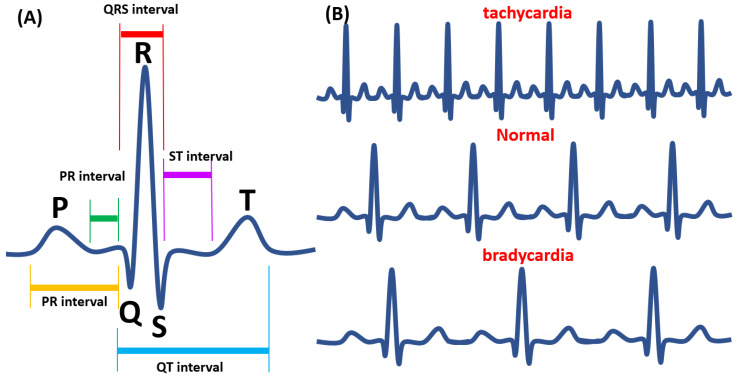
The Electrocardiography (ECG) waveform in adult zebrafish. (**A**) The waveform analysis of ECG. (**B**) Tachycardia (upper panel), normal (middle panel), and bradycardia (lower panel) heart pattern was shown according to a previous publication [73].

Similar to humans’ experience, the different placement of the electrode in adult zebrafish ECGs is thought to account for the variations in T wave morphology and amplitude between different fish. Liu et al. [54] recorded a zebrafish ECG in the presence of hyperkalemia and found the similarity with human ECG during hyperkalemia. They also investigated the change in ventricular repolarization during heart regeneration in an amputation and cryoinjury model and a zebrafish mutant as a human long QT syndrome model. Moreover, in the context of the cardiac action potential in zebrafish, ECG shows similarity with the human cardiac action potential [76]. The anatomy of human ECG and zebrafish is shown in Figure 2.

In adult zebrafish, the technique of needle electrodes is used with the anesthetic model incorporated [76,77]. Anesthetic drugs are commonly used in animal model assay before the experiment is conducted. However, anesthetic drugs were reported to affect the adult zebrafish’s circulatory system and cardiac physiology. A previous study has reported that MS-222 could induce bradycardia. However, isoproterenol, other anesthetic drugs, could increase the heart rate of the fish. The injection of MS-222 caused a heart rate reduction from 160 to 130 beats per minute in one minute. Afterward, injection with isoproterenol has increased the heart rate from 130 to 155 beats per minute. Therefore, the combinational effect of those two anesthetic drugs was studied, and less heart rate alteration was observed. Afterward, in 10 minutes, the heart rate dropped to 64 beats per minute after exposure with MS-222. On the other hand, the heart rate only dropped from 148 to 131 bpm after exposure to MS-222 and Isoflurane combination. In addition, most of the zebrafish in the MS-222 treated group did not recover after sedation. This result suggested that a combination of anesthetic drugs is safer for sedating the zebrafish [68].

Aftereffect of different anesthetic drugs could be observed using the ECG system. A new approach by using other drugs was then performed to see the alteration on cardiovascular performances. Isoproterenol, the agonist of the beta-adrenergic, an amine analog of adrenaline [78], has been widely used for pharmaceutical purposes and has been prescribed to people who have bradycardia [48], as it is known to increase the heart rate of patients. In fish, it is reported that the result was dose-dependent. A dose of 5 µM of the drug caused an increment of heart rate by 1.25-fold. However, in low concentration, 0.5 µM, the recorded heart rate could only increase by 1.04-fold [79].

On the other hand, Mousavi and the colleagues used a recording chamber with a high-resolution camera mounted on the underside coupled with automated recording, heart detection, movement correction, and advanced noise reduction algorithms could allow monitoring the heart rate of swimming fish in real-time without the need for anesthesia [61]. Lauridsen et al.’s findings also supported this study, demonstrating the possibility of measuring heart rate without masking its frequencies even with rapid fish movements [80].

## 12. Light-Cardiogram Methods

Although the body-surface ECG recording method has been proposed in adult zebrafish, it remained inaccessible to other scientists due to the expensive specialized devices and software, low signal-to-noise ratio. In addition, electrode placement’s subjectivity often requires specialist expertise and not readily transferable to other laboratories for routine assessment [81]. Mousavi and Patil established a simple, non-invasive, and inexpensive light-cardiogram technique to assess heart rate and frequency in adult zebrafish. A Bright-field microscope equipped with a high-resolution camera and ImageJ software was used for recording and processing. Collectively, the technique can measure heartbeats and record relative cardiac outputs and compare differences between physiological states (e.g., sexes). Isosceles triangle, as the region of interests (ROI), was placed between opercula and the perpendicular ventral midline. The dynamic pixel change method from ImageJ software was used for heartbeat measurement. The cardiograms generated reverse light signal oscillations. Contraction decreased average brightness within the corresponding ROI; conversely, relaxation increased average brightness. The heartbeat detected by this method was 120 bpm (male 122.58 ± 2.15 and female 121.37 ± 2.63 beats/min), as it is comparable with ECG recording [61]. The approach could be amenable to automation and applicable to other fish species.

## 13. Echocardiography-Based Detection Methods

Echocardiography, which makes use of ultrasound imaging method, has recently been explored to capture the heart image of adult zebrafish. The ultrasound energy is applied to the body through a transducer with piezo-electric transmit and receive ultrasound crystals [82]. A conventional ultrasound imaging device at 8.5 MHz was used to image adult zebrafish hearts [55]. Single-element transducers, a 30-MHz high-frequency ultrasound array system with duplex imaging, were used to measure adult zebrafish hearts and blood flow velocities. Recently, a dual-pulsed wave Doppler imaging method that acquired both spectral and tissue Doppler waveforms simultaneously was developed to enable real-time measurement of the functional change during heart regeneration and adult cardiac structure zebrafish [64]. Most clinical ultrasound scanners can acquire B-mode (brightness) images, M-mode (motion) images, and Doppler (velocity) images. In B-mode imaging, a 2D sector scan is used to create an image where pixel brightness in the image is proportional to the received echo signal’s strength. In M-mode imaging, a 2D image is formed where one image axis is the transducer’s distance, and the other axis is time. As with B-mode, pixel intensities in the M-mode image are proportional to the received echo signal’s strength. M-mode images can be used to track myocardial wall and valve motion. Doppler imaging uses the frequency shift in the received signal to estimate the velocity of ultrasound scatters. Doppler imaging can measure wall and valve motion and assess blood flow through the arteries and heart [82]. In a previous study, two-chamber B-mode images were used to obtain cardiac functional parameters measurement in adult zebrafish, such as stroke volume (SV), ejection fraction (EF), fractional shortening (FS), and cardiac output (CO). Ordinarily, in clinical echocardiography, an image captured from the parasternal short-axis in M-mode would be the most accurate, thus measurement of fractional shortening is the best choice. However, the small size of the zebrafish’s heart (~1.5 mm) makes the M-mode image not easily obtainable [52]. However, other research showed that M-mode imaging was used to analyze the blood flow and tissue motion velocity within the ventricle [55]. High-frequency echocardiography (HFE) has recently allowed the study of cardiac rates in adult zebrafish in 6–9 months old with a body length of ≥ 20 mm. Nevertheless, the image quality was sex-dependent. The adult female zebrafish often was compromised compared to males due to gravidity in females [65].

## 14. Magnetic Resonance Imaging (MRI)

Echocardiography is an inexpensive and widely available technique, but it usually provides only one- or two-dimensional information and is limited to morphological and functional investigations. CT (Computed Tomography) can provide excellent spatial resolution, but relies on ionizing radiation and the application of dedicated stains to achieve soft-tissue contrast. On the other hand, MRI is a well-established clinical diagnostic imaging as it provides multi-parametric information on the heart radiation-free and non-invasively. MRI also is frequently used for in vivo imaging in non-aquatic animal models of cardiovascular disease, including small (e.g., rodents) and large (e.g., sheep, pigs) mammals. Previously, in vivo MRI has been applied to image early developmental stages of frog embryos [56]. The small size of adult zebrafish and optically non-transparent bodies has been challenging to imagine at sufficient resolution. MRM (Magnetic Resonance Microscopy) is established with the same principle as MRI but produces images with a higher resolution because it uses stronger magnetic field gradients (200–1000 mT/m) and specialized radio frequency (RF) coils. Combining high-resolution MRM with 3D image reconstruction makes possible image acquisition of a live adult fish non-invasively, which is impossible using other imaging techniques [57].

Another study by Koth et al. [56] combined cutting-edge technical developments in MRI, novel data processing approaches, and 3D-printing for resolving minimal changes in vivo during zebrafish heart regeneration. To validate the method, they used investigated uninjured, sham-injured, cryo-injured, and resection-injured Tg(*hsp70l:dnfgfr1-EGFP*) transgenic fish which can be conditionally activated dominant-negative *fgfr1* signaling after heat shock treatment. They were able to scan live adult zebrafish under anesthesia and physiological conditions for several hours and with a 100% recovery rate. Thus, they were able to image the same fish repeatedly during the repair process.

The limitation of this method is the small size of zebrafish, in which additional precautions and high-resolution micro-imaging magnet are required to get a good resolution. It has limited space for a flow-through chamber for imaging. The small flow-through chamber cannot support the high water flow, which is needed if noise from surrounding water is to be avoided. Furthermore, zebrafish cannot tolerate a high flow of water. The experimental time should be kept as short as possible since the zebrafish has a relatively low tolerance to anesthetic [57].

## 15. Remote Monitoring Methods

Cardiovascular performances in big fish could also be performed by remote monitoring [58,59]. Modern electronic sensor tags (Bio-loggers) can collect high-resolution data on movement and physiological fish. Electronic sensor tags measure depth, temperature, fluorescence, heart rate, swimming speed, stroke frequency, heat flux, muscle oxygen, acceleration, and many other parameters. Some of these parameters can be used to quantify feeding events and predator-prey interactions. Bio-loggers are often used to measure cardiac performance in big fish. The loggers were surgically inserted into the fish, where the heart’s location provided substantially higher data quality than when the logger was positioned in the fish belly [58]. However, the loggers’ application is considered difficult due to the surgical operation; the cord-based bio-electrodes are invasive and may increase stress to the fish [83]. Recently, new cordless heart rate bio-loggers, which are easier to the implant, were introduced on the market to provide easier tag-deployment and surgery while inducing less stress for the fish [58].

Bio-logging and bio-telemetric devices were commonly used to study stress physiology. The alteration of heart rate and other cardiovascular variables, mostly affected by behavioral responses and environmental perturbations, has been widely used to determine and quantify acute stress responses of fish. Another early physiological indicator of stress; and potential method for assessing farmed fish’s welfare is the changes in gastrointestinal blood flow (GBF). GBF is known to decrease dramatically in fish in response to acute stress, as blood flow is prioritized for oxygen demanding organs and muscle tissues. A previous study has positioned bio-loggers within the abdominal cavity. GBF was measured from the coeliac mesenteric artery, the first caudal branch of the dorsal aorta that divides progressively to supply the stomach, intestine, liver, and gonads [59]. The major disadvantage of bio-logger is the requirement of invasive surgery, as precise placement of tags is required to have high featured data. Generally, when studying fish in the wild, there is a need to recapture the fish to collect the data [84]. Nevertheless, this method is still applicableto big fish, and the application on zebrafish remains unexplored.

## 16. Conclusions

This is the first review of recent and advanced cardiac rhythm detection methods in adult and embryonic stages of zebrafish (methods are summarized in Figure 1. All the proposed methods in detecting heart rate in zebrafish have some advantages and limitations. The heartbeat detection in the zebrafish embryo was mostly performed by a dynamic pixel change method. This method could be performed by open source and user-friendly software, Image-J, or automatically common software Matlab. A laser-scanning confocal microscope is another approach that has been proved to be a sensitive and precision tool for measuring cardiovascular changes in the zebrafish embryo. However, this method is only applicable for transgenic embryos, and it requires costly equipment. While electrocardiography (ECG) is not easy to be conducted in zebrafish embryos, it has been widely used as a gold standard method for the heartbeat and cardiac rhythm detection in adult zebrafish. It is reported that the waveform in adult zebrafish is comparable with the waveform presents in humans. High-frequency ultrasound imaging has also been proposed as a suitable tool for acquiring high-resolution imaging of adult zebrafish tissue structures. Modern electronic sensor tags were established to collect high-resolution data on movement and physiological fish. The loggers were surgically inserted into the fish, close to the heart. However, it is considered difficult due to the surgical procedure and might cause stress on the fish. In conclusion, the cardiac rhythm can be measured at either embryonic or adult zebrafish by utilizing the methods mentioned above. However, the embryonic stage can be better to study cardiovascular function due to its body transparency, independent of anesthetic drugs, facilitate high-throughput heart assay, can be used to detect heart developmental process, and the ability to survive for several days without circulation. The methodology to perform heartbeat and other cardiovascular function parameters can be chosen based on each laboratory capacity. Overall, zebrafish have proven a promising research model for human cardiovascular diseases, pharmaceutical, and toxicological research.

## Figures and Tables

**Table 1:** Comparison of the methods for cardiac rhythm detection in zebrafish embryos.

Author and Published Year	Require a Transgenic Fish Line?	Require a Special Script to Run the Software?	Major Platform to Calculate Heartbeat Regularity	Major Facility to Capture Heartbeat Images	Region of Interests (ROI)	What Kind of Message Can Be Obtained?	Automated Calculation?	References
**ImageJ-Based Methods**								
(Santoso, Sampurna et al., 2019)	No	No	ImageJ (dynamic pixel changes method)	Inverted microscope mounted with high-speed CCD camera	Dorsal Aorta	Atrium rhythm and heartbeat frequency	No	[27]
(Sampurna, Audira et al. 2018)	No	No	ImageJ (dynamic pixel changes method)	CCD mount onto dissecting microscope	Heart	Atrium and ventricle rhythm, whole heartbeat frequency	No	[28]
(Gaur, Pullaguri et al. 2018)	No	No	ImageJ (pixel intensity changes method)	An inverted microscope (Olympus IX73 series) equipped with a 10-MP camera (ProCAM HS-10 MP)	Heart	Whole heartbeat frequency	No	[20]
(van Opbergen, Koopman et al., 2018)	Yes	Not mentioned	ImageJ and MATLAB (method not mentioned)	Upright widefield microscope (Cairn research, Kent, UK) with a high-speed camera (AndorZyla 4.2 plus sCMOS)	Heart	Whole heartbeat frequency	Yes	[29]
**Matlab-Based Methods**								
(Gierten, Pylatiuk et al., 2019)	Yes	Yes	MATLAB (dynamic pixel changes method)	An ACQUIFER wide-field high content screening microscope equipped with a white LED array	Heart	Whole heartbeat frequency	Yes	[24]
(Akerberg, Burns, et al., 2019)	No	Yes	Matlab (deep learning platform)	ZEISS Lightsheet Z.1 microscope and optical microscope	Heart	Whole heartbeat frequency, Cardiac Function Parameters	Yes	[30]
(Xing, Huynh, et al., 2018)	No	Yes	Matlab	Electrophysiological recordings by an Opticam attached to the microscope	Heart	Whole heartbeat frequency	Yes	[31]
(Krishna, Chatti, et al., 2017)	No	Yes	Matlab	Zeiss Stereo Discovery V8 zoom stereo microscope with a ProRes color camera C3 (3 Megapixels)	Heart	Whole heartbeat frequency	Yes	[25]
(De Luca, Zaccaria et al., 2014)	Yes	Yes	MATLAB (threshold value method)	Leica TCS SP5X II confocal laser-scanning inverted microscope equipped with a tandem scanning system	Heart	Atrium and ventricle rhythm, whole heartbeat frequency	Yes	[18]
(Pylatiuk, Sanchez, et al., 2014)	Yes	Yes	MATLAB (threshold value method)	An inverted microscope (Leica DMIL LED) with a digital camera (Leica DFC 400)	Heart	Whole heartbeat frequency	Yes	[26]
(Fink, Callol-Massot et al., 2009)	No	Yes	MATLAB	Hamamatsu EM-CCD digital camera mounted on Leica DM-LFSA microscope	Heart	Atrium and ventricle rhythm, whole heartbeat frequency	Yes	[21]
**Commercial Software-Based Methods**								
(Martin, Tennant, et al., 2019)	No	Yes	FishRateZ software (commercial) (pixel intensity changes method)	AndorZyla 4.2 sCMOS (Andor Technologies, Belfast, NI, UK) camera mounted to a Nikon Ti microscope (Nikon Instruments, Melville, NY, USA)	Heart	Whole heartbeat frequency	Yes	[32]
(Yozzo, Isales, et al., 2013)	Yes	Yes	MetaXpress 4.0.0.24 software (commercial)	ImageXpress Micro (IXM) Widefield High-Content Screening	Heart	Whole heartbeat frequency	Yes	[33]
(Chan, Lin, et al., 2009)	No	Yes	A custom-made program which developed in C# language was used for digital motion analysis	Stereo-microscope (Olympus) equipped with a 3-color CCD camera	Caudal blood vessel	Whole heartbeat frequency	Yes	[34]
(Lin, S.J. 2016)	No	Yes	SoftEdge^™^ (IonOptix Corporation)	Light microscope (Axiovert 100 V microscope, Carl Zeiss, Jena, Germany)	Heart	Whole heartbeat frequency	Yes	[35]
(Ocorr, Fink, et al., 2009)	No	Yes	Semi-automatic Optical Heartbeat Analysis (SOHA)	Not mentioned	Heart	Whole heartbeat frequency, heart diameter measurements	Yes	[22]
Zgenebio and RasVector Technology	No	No	Rv Visual Pulse Analysis	CCD mount onto dissecting microscope	Heart	Atrium and ventricle rhythm	Yes	(https://www.zgenebio.com.tw)
Viewpoint Company	No	No	MicroZebraLab	Not mentioned	Heart	Whole heartbeat frequency	Yes	(http://www.viewpoint.fr/en/home)
Noldus Company	No	No	Danioscope Software	ZEISS SteREO Discovery.V8 microscope	Heart	Whole heartbeat frequency	Yes	(https://www.noldus.com/danioscope)

**Table 2:** Comparison of the methods for cardiac rhythm detection in adult zebrafish.

Author and Published Years	Require Special Transgenic Fish Lines?	Require Special Script to Run the Software?	Major Platform to Calculate Heartbeat Regularity	Major Facility to Capture Heartbeat Images	Region of Interests (ROI)	What Kind of Message Can Be Obtained?	Automated Calculation?	References
**Electrocardiogram-Based Methods**								
Mousavi & Patil (2020)	No	No	ImageJ	Stereoscope (MZ12.5, Leica Microsystems, Wetzlar, Germany) and camera (Dino-Eye Edge series AM7025X)	Whole heart	Heart rate, dominant frequency	No	[61]
Lenning, et al. (2017)	No	Yes	LabView	4-channel MEA membranes for ECG acquisition	Whole heart	Whole heartbeat frequency	Yes	[51]
Vaz da Silva et al. (2017)	Yes	Yes	LabChart program	ECG signals a NeuroLog System	Whole heart	Whole heartbeat frequency	Yes	[62]
Liu et al. (2016)	No	Yes	Clampfit 10.0 software	ECG recording	Whole heart	Heart rate, PR, QRS, and QT intervals	Yes	[54]
Chaudari et al. (2013)	No	Yes	Power lab software	ECG recording	Whole heart	Heart rate, QT, PR and RR intervals	Yes	[63]
**Echocardiography-Based Detection Methods**								
Chiang, et al. (2020)	No	Yes	Matlab	A 70-MHz ultrasound imaging system and single-element transducer	Heart and dorsal aorta	Blood flow, tissue velocity, and cardiac deformation measurement	Yes	[55]
Yeo, Yoon et al. (2019)	No	Yes	Matlab	Custom-built, 64-channel high-frequency array imaging system and a high-frequency linear array transducer with 256 elements	Heart and dorsal aorta	Blood flow velocity, Heart regeneration	Yes	[64]
Wang, Huttneer et al. (2017)	Yes	Yes	Vevo Lab™ analysis software	Vevo2100^®^ Imaging System and Vevo Imaging Station (VisualSonics) equipped with a high frequency transducer	Whole heart	Cardiovascular function parameters	Yes	[65]
Lee, Genge et al. (2016)	No	Yes	VisualSonics software	Vevo 2100 ultrasound system (VisualSonics1, Toronto, ON, Canada), with a 70 MHz ultrasound transducer	Whole heart	Heart rate, stroke volume (SV), ejection fraction (EF), fractional shorting (FS) and cardiac output (CO)	Yes	[52]
**Magnetic Resonance Imaging (MRI)**								
Koth, Maguire, et al. (2017)	Yes	Yes	Matlab	MR scanner	Whole heart	Heart regeneration	Yes	[56]
Kabli, Alia et al. (2006)	No	Yes	Para Vision 3.02pl running on a Silicon Graphics 02 workstation with the Irix 6.5.3 operating system and using Linux XWinNMR 3.2	Magnetic Resonance Microscopy (MRM) consist of microimaging probe, MR magnet	Whole body	Developmental process in zebrafish	Yes	[57]
**Remote Monitoring Methods**								
Norling (2017)	No	Yes	Software AcceleRater (python-based web application)	DST micro-HRT logger	Whole heart	Heart rate, Behavior performance	Yes	[58]
Brijs, Sandblom et al. (2019)	No	Yes	LabChart Pro software	Custom-built Logger	Heart and coeliacomesenteric artery	Blood flow, Heart rate	Yes	[59]
